# Examination of the role of magnetic resonance imaging in multiple sclerosis: A problem-orientated approach

**DOI:** 10.4103/0972-2327.58284

**Published:** 2009

**Authors:** Henry F. McFarland

**Affiliations:** Neuroimmunology Branch, NINDS, NIH, Bethesda, USA

**Keywords:** Magnetic resonance imaging, multiple sclerosis, problem-oriented clinical approach

## Abstract

Magnetic Resonance Imaging (MRI) has brought in several benefits to the study of Multiple Sclerosis (MS). It provides accurate measurement of disease activity, facilitates precise diagnosis, and aid in the assessment of newer therapies. The imaging guidelines for MS are broadly divided in to approaches for imaging patients with suspected MS or clinically isolated syndromes (CIS) or for monitoring patients with established MS. In this review, the technical aspects of MR imaging for MS are briefly discussed. The imaging process need to capture the twin aspects of acute MS viz. the autoimmune acute inflammatory process and the neurodegenerative process. Gadolinium enhanced MRI can identify acute inflammatory lesions precisely. The commonly applied MRI marker of disease progression is brain atrophy. Whole brain magnetization Transfer Ratio (MTR) and Magnetic Resonance Spectroscopy (MRS) are two other techniques use to monitor disease progression. A variety of imaging techniques such as Double Inversion Recovery (DIR), Spoiled Gradient Recalled (SPGR) acquisition, and Fluid Attenuated Inversion Recovery (FLAIR) have been utilized to study the cortical changes in MS. MRI is now extensively used in the Phase I, II and III clinical trials of new therapies. As the technical aspects of MRI advance rapidly, and higher field strengths become available, it is hoped that the impact of MRI on our understanding of MS will be even more profound in the next decade.

## Introduction

Over the past two decades magnetic resonance imaging (MRI) has substantially altered our ability to study and manage multiple sclerosis (MS). First, it has provided a relatively accurate measure of disease activity; second, it has enhanced our ability to diagnose the disease, thus allowing earlier treatment; and third, it has provided a powerful tool to study the benefits of new therapies. Despite these advances, many questions remain regarding the relationship between what is seen on MRI and the clinical course of the patient. This review will attempt to explore the strengths and weaknesses of MRI in the care of patients with MS.

## Technical Aspects of Applying MRI to MS

The usefulness of MRI in MS will be only as good as the quality of the images. The usual goals of imaging in MS include the following: to establish that the pattern of disease is consistent with what is expected in MS and does not suggest some alternative diagnosis, to see if the MRI findings are consistent with the MRI criteria now incorporated into the diagnostic criteria for the disease, and to assess the level of activity of the disease which, in turn, can contribute to treatment decisions. In 2001 and 2003 the Consortium of Multiple Sclerosis Centers CMSC (CMSC.org) presented imaging guidelines recommended by a panel of experts in the field. These guidelines have now been revised and are available on the CMSC web site. The guidelines are divided into approaches for imaging of patients with suspected MS or clinically isolated syndromes (CIS) or for monitoring patients with established MS. With respect to imaging suspected cases, several important points need to be stressed. The MRI should be done with contrast. Not only does this help improve diagnostic specificity but it also allows some assessment of activity. Second, if spinal cord symptoms are suspected, the spinal cord should be imaged. No specific recommendations are made with respect to field strength since, in any case, MRI centers are now generally equipped with either 1.5- or 3-Tesla units. As will be discussed later in this chapter, every effort should be made to use a standard imaging protocol since that will allow comparison of images done at different time periods. As noted in the CMSC recommendations, the core sequences will be a sagittal FLAIR, axial FLAIR, axial T2, and axial T1 pre- and post-gadolinium imaging. All of these sequences should be done with 3-mm slices and no gap.

The application of MRI in periodic monitoring of patients is more complex and there is some disagreement among MS experts regarding this use. The difficulties of using MRI to monitor the effectiveness of a therapy in clinical practice are considerable. Both are discussed later in this review

## Measuring Acute Disease using MRI

**Figure 1:**
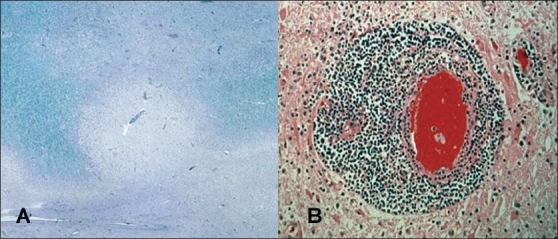
Pathological specimen from a patient who died from an acute worsening of MS. A contrast enhanced MRI done one week prior to death showed enhancing lesions. Histological specimens from corresponding area of the brain showing acute demyelination (Fig 1A) and inflammatory changes (Fig 1B).

Currently, it is thought that MS consists of two components. The first is characterized by acute inflammation and is thought to be the result of an autoimmune process. The second component is a neurodegenerative process. The relationship between the two is debated but, most likely, inflammation sets the stage for later neurodegenerative processes. The greatest success with MRI to date has been in the identification of acute inflammatory disease. New lesions seen on T2-weighted or FLAIR images represent lesions that are almost certainly secondary to focal inflammation. More specifically, imaging done after the administration of a contrast agent such as gadolinium can identify these acute inflammatory lesions with great accuracy. Early in the application of MRI to MS, evidence emerged showing that contrast enhancement predicted the appearance of new lesions[1,2] and that contrast enhancement was related to acute inflammation.[3] Figure 1 shows an example of pathology in a patient who died from an episode of acute worsening of her MS. The patient had been studied with a contrast-enhanced MRI a little over a week before her death. The pathology shown in Figure 1 is from the area of the brain that enhanced on the MRI 3.

Considerable success has been achieved using postcontrast T1-weighted MRI to study the natural history of MS as well as to measure the effectiveness of therapies targeting the early, inflammatory aspect of MS.[4–6] The use of contrast-enhancing lesions (CELs) as an outcome has proved invaluable in phase I and early phase II clinical trials to establish preliminary evidence of the effect of the therapy and to justify phase III trials of a therapy, as these trials are costly both in dollars as well as in human resources. A major concern exists, however, with regard to the understanding of the significance of CELs. Multiple studies have examined the relationship between enhancing lesions and relapses and all have found that though a relationship exists, it is weak.[7,8] A recent study has explored the extent to which CELs could meet the criteria for a validated surrogate for relapses.[9] The ability to use CELs as a surrogate would be valuable for shortening clinical trials but, unfortunately, CELs failed to meet the formal criteria for validating a biomarker as a surrogate. Some studies have suggested a modest relationship between CELs and disability over short periods of time,[10,11] but an analysis of the relationship of the average number of CELs on three serial MRIs and disability (on average) 8 years later failed to show any relationship with disability (Stone *et al*. submitted). In contrast, a long-term follow-up study of a cohort of patients presenting with a clinically isolated syndrome (CIS) has shown a relationship between the number of lesions seen on a T2-weighted image and disability 20 years later.[12] Since it is generally held that most if not all CELs persist as lesions on T2-weighted images and that essentially all lesions seen on T2-weighted images beginning with blood–brain barrier (BBB) breakdown, the lack of a relationship between CELs and disability becomes difficult to explain. The modest relationship between CELs and short-term disability or conversion to Clinically Definite Multiple Sclerosis (CDMS) (discussed later in this review) is most likely related to the relationship of CELs to relapses. The disconnect between enhancing lesions and disability in the long term suggests that processes other than just evidence of disruption of the BBB are contributing to disability. Several explanations may contribute to the lack of correlation between CELs and disability. Location of the lesion is certainly critical and it is well understood that some lesions such as those in the spinal cord contribute disproportionally to disability, especially as measured by the Expanded Disability Status Scale (EDSS). Yet it would be expected that lesions in the cord would be more frequent in patients with a higher frequency of enhancing lesions and that a relationship between the number of CELS and disability would exist. A second possibility is that not all enhancing lesions are the same and, in fact, considerable evidence for heterogeneity exists. Also, it is now well documented that damage in white matter distal to the lesions, the so-called normal-appearing white matter (NAWM), contributes to disability. Finally, disease activity involving the gray matter no doubt contributes to disability. Each of these possibilities will be discussed below.

### Are all acute inflammatory lesions the same?

**Figure 2:**
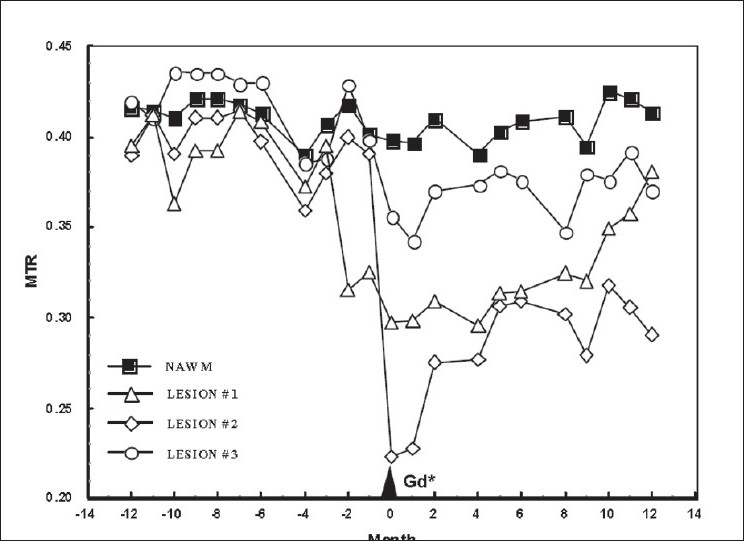
Time course of MTR changes in two enhancing lesions using serial, registered images. Differences in MTR changes and recovery noted.[31]

It is now clear that all CELs are not the same; some are associated with greater destruction than others. While essentially all lesions seen on a T2-weighted image begin as contrast-enhancing lesions, variability is evident using other imaging techniques such as magnetization transfer and diffusion-weighted imaging or using the appearance on noncontrast T1-weighted images. Some, but not all, acute lesions will remain or become hypointense on precontrast T1-weighted images (black holes).[13] Acute CELs that enhance for longer periods of time when followed using serial monthly MRIs are more likely to become persistent black holes,[14] suggesting that either a qualitative or quantitative difference exists in the inflammatory response between those lesions that become black holes *vs* those that do not. A second technique used to demonstrate lesion heterogeneity is magnetization transfer imaging. The results are expressed as a ratio – the magnetization transfer ratio (MTR). MTR imaging depends on the exchange of protons between a macromolecular structure and water. The macromolecular content of tissue can be indirectly measured by using two images; an off-resonance pulse to saturate bound protons associated with a macromolecular structure will affect the magnetic exchange with free-water protons and alter the signal from the free-water pool. The results are expressed as the signal intensity, with and without the saturation pulse. A decrease in MTR indicates a probable decrease in the macromolecular structure. Since, in white matter, the macromolecular structure that is rich in protons is predominantly myelin, MTR is thought to represent, predominantly, a measure of myelin integrity.[15] Clearly, however, MTR is sensitive to loss of axons. A strong correlation has been shown between black holes, the decrease in MTR, and axonal loss in tissue samples.[16] A number of investigators have reported differing evolutions of MTR in new CELs.[17,18] Figure 2 shows serial MTR values for three regions of interest (ROIs) that will become CELs at one time point.

The actual pathological substrate for the recovery of MTR in lesions is uncertain and could represent decreasing inflammation, remyelination, or both. The interpretation of the heterogeneity of MTR recovery of lesions is complex. Most likely, both the magnitude of the inflammatory response and the level of tissue destruction are reflected in the MTR. The important point is that those lesions which persist with reduced MTR values probably have the greatest levels of myelin and axonal loss. The association between decreased MTR and axonal loss has been demonstrated in a study examining the pathological changes seen in lesions that persist as black holes.[16] Using tissue samples from patients who have died from MS, a relationship between the degree of hypointensity on T1-weighted images and the MTR within these lesions has been shown to correlate with axonal density.

**Figure 3:**
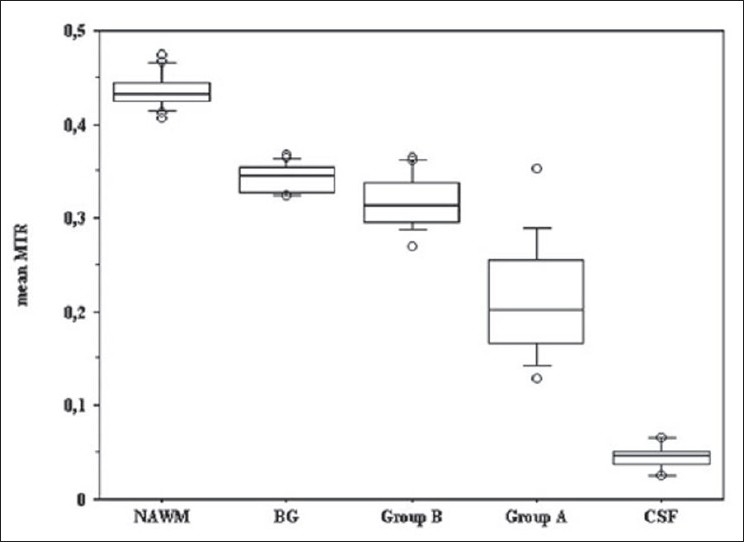
Differences in MTR values between type 1 and type 2 lesions using tissue specific imaging[66]

Similar evidence has evolved from studies using diffusion tensor imaging (DTI). Two primary diffusion measurements can be made. The first represents the diffusion of protons along a confined longitudinal space [fractional anisotropy (FA)] and the second is radial diffusion, as would occur in free water (diffusivity). Again, uncertainty exists as to the relative contributions of myelin and the axon to FA; both no doubt contribute. Regardless, the results indicate that as the degree of tissue destruction increases, FA will decrease and diffusivity will increase.[19] Again, using this technique, results indicate differences in the level of tissue destruction.[20,21] Recently, a technique termed Tissue Specific Imaging (TSI) had been developed. TSI allows selective imaging of the three tissue compartments in the brain – i.e., white matter, gray matter, and CSF –has been applied to studying heterogeneity of lesions.[22] Using this approach, a small number of lesions can be identified that have signal intensity identical to CSF. Characterization of these lesions using MTR and DTI indicates that these lesions represent those with the greatest degree of tissue destruction. The differences in Fractional Anisotropy (FA) and Mean Diffusivity (MD) between lesions that are seen as hypointense on a T1-weighted image and on the TSI image (group B) and those that are seen only on the T1-weighted image (group A) are shown in Figure 3.

It is also worth noting that new contrast agents have been applied to the study of MS and results indicate that varying levels of lesion development are now being identified.[23–25]

All of the evidence described above indicates that considerable differences exist with respect to the level of tissue damage following the appearance of a CEL. Thus, it is likely that a part of the disconnect between the frequency of CELs and future disability is related to the level of tissue destruction that remains following the initial lesion

## Using MRI to monitor progressive disease in MS

The use of MRI to monitor the progressive phase of MS has seen less success than has been achieved in the acute inflammatory phase. The ability to find MRI markers that correlate well with progression of disability has been difficult. The most commonly used metric is brain atrophy. Various techniques have been used to measure atrophy but an analysis of the relative merits of these various techniques is beyond the scope of this review. Generally, all have reported about 1% loss of tissue per year compared to a loss of about 0.1% in healthy individuals. Brain atrophy is currently the most extensively studied of all of the global measures of tissue damage and is currently being used as an outcome in trials examining neuroprotective strategies.[26] Other global measures that have been applied are whole-brain MTR and whole-brain Magnetic Resonance Spectroscopy (MRS).[27,28] However, most investigators now analyze tissue segmented into gray matter and NAWM.[29] Findings from the studies cited above, along with that from many others, suggest that damage removed from lesions that are demonstrated by conventional MRI may contribute to disability. In NAWM, abnormalities have been demonstrated using a number of imaging techniques including MTR, MRS, DTI, and T2 relaxation.[30–35] In MS, evidence supports significant changes in NAWM beginning at an early stage of disease; patients with CIS tend to have identifiable changes in MTR in NAWM as compared to control individuals.[32] Of note, when correlations were sought between MRI measures and conversion from CIS to MS, abnormities in NAWM did not contribute, whereas the number of lesions seen on T2-weighted images did. The findings are consistent with the idea that focal lesions have the strongest relationship to relapse, which is necessary for clinical conversion to MS from CIS. What remains unclear is whether the abnormalities in NAWM are a result of an acute focal lesion or represent a partially independent process. Studies of the pathology of MS have described the presence of immune cells in NAWM,[36,37] and diffuse inflammation could represent the substrate for the development of acute lesions. Consistent with this hypothesis is the finding that abnormalities in MTR may predict the development of an acute lesion many months in advance.[38,39] An example of decreased MTR preceding the development of acute lesions can be seen in Figure 2.[18] In contrast, analysis of the magnitude of damage in NAWM surrounding lesions indicate that the damage is greatest close to the lesion and diminishes with distance from the lesion.[40] Consequently, the processes responsible for the abnormalities in NAWM are not well understood but no doubt are critical for our understanding of the disease. As will be discussed below, considerable evidence now exists for involvement of gray matter. It is possible that neuronal damage resulting in structural or metabolic impairment of axons in the white matter could contribute to focal alterations in axon-myelin integrity.

### Involvement of gray matter

Involvement of gray matter in MS has now been documented both by histological studies as well as by MRI. Lesions in the cortex are seen on pathological examination and can be seen on MRI also. The cortical lesions have been classified based on appearance and extent of involvement and on whether the lesion involves white matter as well as gray matter.[41,42] Included in the classification are subpial lesions involving the first two layers of the cortex, lesions that are entirely confined to the cortex but may involve all layers of the cortex, and lesions that involve both gray and white matter. Several characteristics of the pathology of cortical lesions are important to note in considering the ability to identify these lesions using MRI. Lesions within the cortex generally have relatively small amounts of inflammation though demyelination is present. Of note, lesions that involve both gray and white matter have been shown to have the characteristic acute inflammatory characteristics of MS in the white matter portion but only limited inflammation within the gray matter portion of the lesion.[42] The results suggest that processes within the brain that contribute to amplification of inflammation may differ between gray and white matter. Only occasionally are contrast-enhancing lesions seen in the cortex. Demonstration of cortical lesions by MRI has received considerable interest in the past few years but has met with limited success.[43,44] A variety of imaging techniques have been used, including Double Inversion Recovery (DIR), Spoiled Gradient Recall (SPGR), and Fluid Attenuation Inversion Recovery (FLAIR).[14,45–47]

In addition to lesions within the cortex, atrophy of both cortical and deep gray matter is well documented in MS.[43] Again, a variety of techniques have been used to measure atrophy. Atrophy of the cortex can be seen early in the disease course and has been reported to be more marked than atrophy of white matter.[48] Of note, atrophy of gray matter appears to be more marked in progressive disease than in Relapsing Remitting Multiple Sclerosis (RRMS).[49] The finding suggests that independent processes may be involved and this hypothesis is consistent with the lack of a correlation between the pathological changes in white matter and those in gray matter.[50] Atrophy of deep gray matter structures is also observed in MS.[51] Changes are more evident in the thalamus than in other components of the basal ganglion. Correlations between atrophy of deep gray matter (including the thalamus and hippocampus) and some measures of cognitive function have been reported.[52,53] Still uncertain is the relationship between gray matter lesions, gray matter atrophy, and disease in white matter.

Currently, a widely accepted concept of MS is that it begins with an inflammatory process but then evolves into a neurodegenerative disease. The relationships between acute lesions and other components of the disease process such as damage in NAWM and in both cortical and deep gray matter have been discussed above. Again, it should be stressed that the relationship between acute inflammation and measures of more diffuse disease remains uncertain. In contrast to measurements of damage to specific tissue compartments, a number of global measures have been used to assess the overall degenerative component of the disease.

## Use of MRI to monitor clinical trials

In addition to being an important biomarker in MS, MRI has also had an important role in advancing new therapies.[54] Since CELs are recognized as reflecting an acute inflammatory stage of disease, they have been used as an outcome for various types of clinical trials. As discussed above, CELs do not meet the criteria for a validated surrogate and therefore cannot be used in place of a clinical outcome in clinical trials that will be used to request registration of the treatment by either the American or European health authorities. However, CELs have been used to assess safety in phase I studies and as an outcome in early phase II studies designed as proof-or-principle studies. Finally, imaging outcomes represent important secondary outcomes in phase III clinical trials and are useful in helping to understand the mechanisms of the therapy.

### Phase I studies

Many of the new therapies in development over the past several years have mechanisms of action that are poorly understood. Consequently, at the initial exposure of a patient with MS to the therapy, there is always the possibility that the therapy may increase rather than decrease disease activity. Since MRI is a very sensitive measure of new inflammatory disease activity, MRI outcomes are effective in monitoring safety. An example is a recent study of a PDE4 inhibitor.[55] Studies in the mouse EAE model indicated that PDE4 inhibition was associated with a decrease in disease activity, both clinically and pathologically. The therapy was shown to decrease Th1 activity and promote Th2 activity. The initial clinical trial of a PDE4 inhibitor, rolipram, was done to examine safety. An initial cohort of patients with no ongoing activity was studied to see if new activity occurred on therapy. Next, a second cohort with ongoing new activity was studied to assess the effect of the therapy on patients with active disease. The results indicated that the therapy most likely increased disease activity and the study was therefore stopped.

### Phase IIa studies (proof-of-principle)

Probably the most effective use of MRI as an outcome has been in early phase II studies. An interesting example is the study of IFNb1b that used a simple cross-over design to examine the effect of IFNb1b on CELs after the drug had completed phase III testing and had been approved for use.[56] The study demonstrated clearly that IFNb1b targeted an early inflammatory stage of disease. Since then, studies with similar designs have been used in proof-of-principle studies of a number of therapies. MRI represents an important outcome in early testing of essentially all of the therapies now undergoing phase III testing.

**Figure 4:**
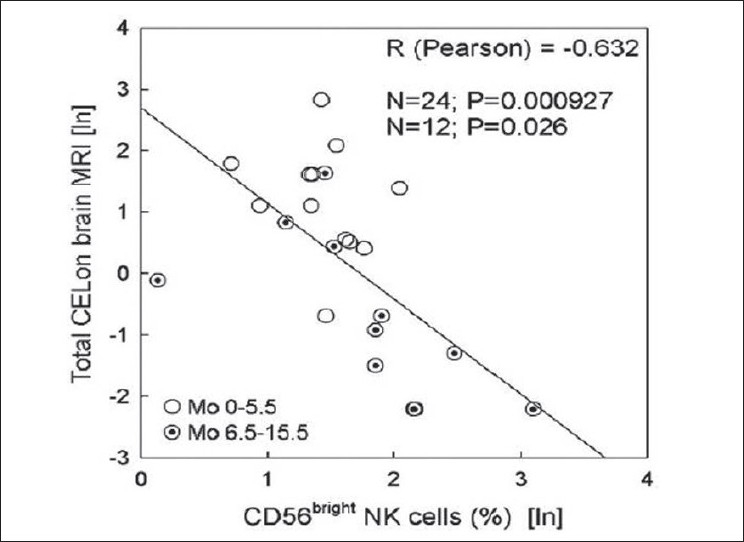
Correlation between expansion of CD56bright NK cells and inhibition of brain inflammatory activity during treatment with daclizumab[58]

A recent study of a monoclonal antibody, daclizumab, that blocks the alpha chain of the high-affinity IL-2 receptor, CD25, has been studied in a series of small clinical trials using MRI as the primary outcome.[57,58] The results of these studies have shown that daclizumab substantially reduces the frequency of CELs. The design used in these studies was similar to that described above for rolipram. In addition to assessing the effect of therapy on the imaging outcome, the effect on the patient's immune response was also interrogated. The immunological studies demonstrated that therapy was associated with an increase in a population of NK cells, which appear to have regulatory function. The combination of the detailed immunology with the imaging outcomes demonstrated a very strong association between the expansion of the NK population and the reduction in the number of CELs while on therapy [Figure 4].

A second example is campath 1H or alemtuzumab.[59] Alemtuzumab is a monoclonal antibody directed at CD52, which is expressed on a number of immunologically active cells. Alemtuzumab therapy results in significant immunosuppression and had been used to treat B-cell chronic lymphocytic leukemia. The initial study of alemtuzumab in MS consisted of treating a relatively small cohort of patients, including those with RRMS and Secondary Progressive Multiple Sclerosis (SPMS) clinical courses. The results demonstrated a substantial reduction in CELs and in T2 lesion load. There was also reduction in relapse frequency. However, many patients, particularly those already in the SPMS phase of disease, continued to have progression of disability and progression of brain atrophy. The results of this study provided one of the clearest demonstrations that progression of disability and brain atrophy can become relatively independent of acute inflammatory activity once the disease is well established.[60] The results support the hypothesis that while MS begins as an acute inflammatory process, disease progression is related to degenerative processes that are relatively independent of the appearance of new inflammation. This hypothesis has been further validated by a subsequent study of alemtuzumab in patients with early MS; in this cohort, a substantial reduction, and even improvement, in disability was reported.[61]

### Phase III studies

MRI represents a secondary outcome in most phase III clinical trials in MS. Since all therapies examined in definitive phase III clinical trials to date have been therapies that target an inflammatory component of disease, evidence that the therapies decrease CELs or accumulation of disease burden have been useful in confirming the effectiveness if the therapy. More important, however, will be the use of advanced imaging techniques to better understand the disease process and the effect of the new therapy on that process. Since many of the new therapies are very effective in reducing inflammation, using advanced imaging techniques that could provide better information on the effect of the therapy on tissue damage will be valuable.

Unfortunately, funding for clinical trials that involve the application of advanced imaging and immunological evaluations is often a problem. Also problematic is the ability to get sponsors of clinical trials to invest in imaging techniques that go beyond the standard measurements of lesion load and inflammation.

## Use of imaging in the diagnosis of MS

After the initial observation that MRI could be used to image lesions in MS, it has found widespread acceptance in the diagnosis of MS. Prior to the use of MRI in helping to establish dissemination in space and time, the diagnosis of MS was often difficult in the early stages of the disease. The initial efforts to formalize MRI criteria in the diagnosis were driven by the use of MRI in clinical trials. Because these various criteria were associated with particular clinical trials, there was a need for criteria that was based on a body of evidence from natural history studies. In 2000, an international panel proposed a set of unified diagnostic criteria incorporating MRI parameters that would establish dissemination in space and time.[62] The criteria was based on a series of natural history studies that had examined MRI findings in patients with CIS that predict definite MS.[63,64] In 2007, the diagnostic criteria was revised and these are outlined in Table 2.[65] Readers are advised to review the entire diagnostic criteria; only the MRI criteria for dissemination in space or time are shown in Table 2.

**Table 1:** Relationship between MTR values and axonal density using postmortem tissue[16]

Lesion Characteristic	MTR	Axon Density %
No T2	0.32	90
T2 Isotropic on T1	0.30	80
Mild T1 hypointensity	0.24	50
Marked T1 hypointensity	0.15	30

**Table 2:** Magnetic resonance imaging criteria for dissemination in time and space[65]

Dissemination in space	Three of the following
	At least 1 CEL or 9 T2 lesions
	At least 1 juxtacortical lesion
	At lease 1 infratentorial lesion
	At lease 3 periventricula lesions
Dissemination in Time	One of the following
	Demonstration of a CEL on a MRI done at least 3 months after the clinical onset and in a location not corresponding to the site of the initial event
	Detection of a new T2 lesion on any scan compared to a reference scan done 3 months after the initial event.

Recently, some investigators have proposed simplified criteria that would allow the diagnosis to be made based on a single MRI done at the time of presentation with an initial clinical event. Hopefully, consensus will be reached quickly on the best approach. It is important to note that the criteria emerging from the international panel placed an emphasis on the specificity of the diagnostic criteria. A goal was to propose criteria that would have the greatest chance of correctly establishing the diagnosis, since the diagnosis of MS carries with it many implications related to, for example, employment and insurance. Proposals for more liberal diagnostic criteria have tended to place emphasis on sensitivity or the ability to diagnose the illness at the earliest stage so that therapy can be started. Although the studies that have supported the more liberal criteria have shown reasonable specificity in addition to sensitivity, they have focused only on patients who have been followed in tertiary MS centers. Consequently, the rate of misdiagnosis in a general neurology clinic is unknown and that is of concern.

## Can MRI be used to monitor individual patients?

**Figure 5:**
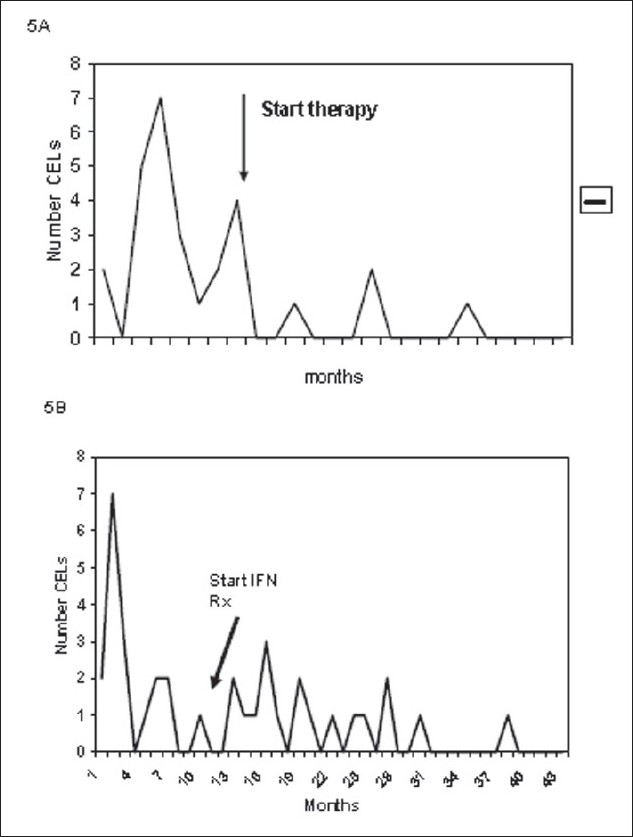
Difficulties in using MRI to monitor treatment response in individual patients

Extrapolation of the successes in using MRI in clinical trials to the care of patients in the clinic, and to study the natural history of MS, has been a constant goal of researchers but one that has proven difficult to reach. Identification of the effectiveness of a therapy in a clinical trial requires either knowledge of the level of disease in an individual patient prior to beginning therapy or the ability to compare the response of a group of patients to matched patients receiving a placebo. Even the cross-over design discussed previously has limitations for interpreting the effect of therapy in an individual patient. Levels of disease activity are extremely variable and the frequency of new lesions tends to decrease with time. Therefore, the use of MRI in the clinic to judge the effectiveness of treatment in an individual patient is associated with a considerable risk of error. For example, on examining the data in Figure 5, it can be seen that if only one MRI had been available pretreatment, the conclusions regarding the effect of therapy would have been very different. The potential error is compounded when the second MRI is taken while on therapy; the timing of the MRI could again lead to different conclusions. The patient shown in Figure 5A is considered to have responded to therapy when the entire pattern of MRIs is examined. However, if individual snapshots are considered, there is a good chance of arriving at the wrong conclusion. The patient in Figure 5B is considered a nonresponder or, at best, a partial responder and, again, snapshot MRIs could fail to capture changes in the magnitude of disease while on therapy.

Consequently, when MRI is used to measure actual disease as an assessment of effectiveness of therapy, it must be done with caution. Clearly, patients having high levels of disease on a therapy known to reduce acute disease activity can be considered to be nonresponders. Low levels of disease or even lack of acute disease are not easily interpreted on a single MRI. Essential in any effort to use MRI to monitor patients is the consistency of the MRI studies. Readers are referred to the proposed guidelines on the CMSC web site.

Are MRI measures other than those that identify acute disease activity valuable in monitoring patients? A number of global measures of disease are often monitored, including T2 lesion load, atrophy, and volume of T1 hypointensities. All of these measures require some level of computer-based image analysis in order to be accurate. For global measures to be helpful, careful attention must be paid to the technical aspects of the images. Essentially, the pretreatment and treatment imaging should be done using similar techniques, which is often not the case when imaging is done outside of a research setting. Finally, there is concern over the interpretation of changes in global measures, since the effect of therapy is less well understood. In summary, any effort to use MRI to monitor the effect of a particular therapy in the clinic must be done with considerable care and the awareness that findings may be difficult to interpret.

For the neurologist outside of a specialized MS clinic it will be important to establish a working relationship with the radiologist or neuroradiologist who will conduct and read the MRI studies. It is important that the radiologist understand the goals of the neurologist and the importance of maintaining consistency in the imaging studies.

## Conclusions

The application of MRI to the study of MS represents one of, if not the, greatest advances in this field. It has lead to a much better understanding of the natural history of this disease. Probably most important in this respect has been the demonstration that the disease is often active during the early phases of the illness, even during periods when the patient is clinically stable. This observation has, in turn, lead to the interest in conducting trials in patients with very early MS and to the treatment of patients – often after the initial neurological event – if MRI findings are consistent with the diagnosis and indicate disease activity.

MRI has also facilitated the conduct of treatment trials, especially early proof-of-principle studies, necessary to commit large amounts of resources to more definitive clinical trials. Unfortunately, some of the initial hopes that MRI would solve most of the questions in MS have not come to be. It is now clear that there is much that we do not understand with respect to the basis of disease progression. On the positive side, MRI has shown us that the disease is more than discrete white matter lesions and it has been responsible for opening many new areas of research into the disease. Finally, as the technical aspects of MRI advance rapidly and higher field strengths become available, it is hoped that the impact of MRI on our understanding of MS will be even more profound in the next decade.
